# Advancing research in adult secure mental health services in England

**DOI:** 10.1177/00258024211066981

**Published:** 2021-12-15

**Authors:** Howard Ryland, Louise Davies, Jeremy Kenney-Herbert, Michael Kingham, Mayura Deshpande

**Affiliations:** 1 105611 Department of Psychiatry, University of Oxford; 2 Adult Secure Clinical Reference Group, NHS England and NHS Improvement; 3 Birmingham and Solihull Mental Health NHS Foundation Trust; 4 Kent and Medway NHS and Social Care Partnership Trust

## Abstract

Forensic mental health services in high income countries are typically high cost and low volume, providing care to people with mental illness, personality disorders, learning disability and autism deemed to pose a risk to others. Research into how forensic mental health services work as a whole system is limited. Such research is urgently needed to guide policy makers and ensure that services operate effectively.

This paper describes an initiative by a commissioning advisory body, the Clinical Reference Group for Adult Secure Services, at NHS England and NHS Improvement, to pioneer a new approach to advancing research for forensic mental health services. This involves the systematic canvasing and prioritisation of research questions from stakeholders, including patients, carers, academics, and clinicians.

The process has already been effectively used to commission research in one priority area, namely evaluating access assessments to low and medium secure services. This entailed positive engagement by all stakeholders and demonstrated the value of the Clinical Reference Group as a nexus for coordinating such activities.

There are however challenges to the delivery of this much needed research, not least the dwindling numbers of clinical academics in the field. Clinical leadership and co-production with experts by experience will be essential to ensure the relevance of research outputs to real world settings.

More systematic mechanisms are needed to identify and initiate research in areas that matter to frontline mental health services. Commissioning organisations offer one potential route to deliver this by linking experts by experience, clinicians, policy makers and commissioners.

## The need for health services research in forensic mental health services

Forensic mental health services provide care and treatment for people with mental illness, personality disorders, learning disability and autism who are also deemed to pose a risk to others.^1^ In high income countries these services are often very expensive, consuming a considerable portion of the resources available for healthcare. For example, in England adult secure inpatient services cost £1.3 billion for approximately 6785 commissioned beds. The average length of stay is long compared to general adult mental health services, with as many as 27% of patients staying over 10 years.^2^ One study in north London estimated that the mean length of stay for patients in forensic beds was 2272.6 days compared with 697.6 for those in general adult beds.^3^ The reasons for this discrepancy are not fully understood but may relate to a shift towards the forensic pathway as the dominant way of accessing longer term placements. Patients in forensic services are also often subject to considerable restrictions during their admission.^4^ After discharge, patients will often continue to be subject to limitations on their liberty and to reporting requirements.^5^ The consequences of recidivism can be severe for victims and their families, as well as for patients themselves.^6^ Provision of forensic mental health services appears to be rising, with increasing capacity in many parts of the world.^7^ For example, in England and Wales the number of patients detained in hospital under a restriction order, which allows the Ministry of Justice direct control over many aspects of their admission and discharge, increased from 3118 in 2003 to 4679 in 2016.^8^ For these reasons it is important that forensic mental health services are working effectively to fulfil their dual purpose of public protection and patient care.

Currently much research in forensic mental health services focuses on certain, well-defined questions, such as establishing the risk factors for violence or the effectiveness of particular interventions. The results of research conducted in other settings, such as randomised controlled trials of the effectiveness of pharmacological and psychological interventions, is often applied to the forensic setting without adequate testing in forensic services. There is limited research in to what makes forensic mental health services operate effectively as a whole.^9^ However, such research is essential to ensure the proper functioning of these services, which are highly complex because they involve the simultaneous delivery of multiple interventions. These interventions typically include a combination of prescribed psychotropic drugs, psychological therapies and occupational therapy.^10^ Furthermore, their success relies on other less tangible factors that may not be considered as discreet interventions in and of themselves, such as the relational security and the therapeutic milieu provided by staff.^11^ Despite barriers to their participation, co-production with patients, and their famies and carers, is very important and they must also be involved in developing and implementing research in this field, to ensure that it aligns with the priorities of the people who use services.^12,13^ Furthermore, academic psychiatry in the UK has witnessed a substantial decline in capacity over recent years. The Medical Schools Council identified a reduction of 84.4 full time equivalent academic posts in psychiatry between 2007 and 2018.^14^ Despite systemic recognition of this problem and initiatives to improve the situation, there remain concerning questions about the ability to deliver the research needed.^15,16^

Health services research is therefore required to evaluate and improve the effectiveness of forensic mental health services, which depend on the many component parts of the system working together.^17^ Mechanisms are needed to systematically identify and prioritise research questions from experts by experience, commissioners and frontline clinical services, to drive this research agenda. This should be coupled to a pathway for subsequently identifying and resourcing researchers to respond to the questions selected, which in turn informs the policy response.

In this paper we describe an initiative undertaken by the Clinical Reference Group (CRG) for Adult Secure Services at NHS England and NHS Improvement (NHSEI) which seeks to pioneer such an approach to advancing research for adult inpatient forensic mental health services. We describe the process and reflect on lessons learned, before setting out a vision for the future.

## Clinical reference group for adult secure services research prioritisation process

**Figure 1.**
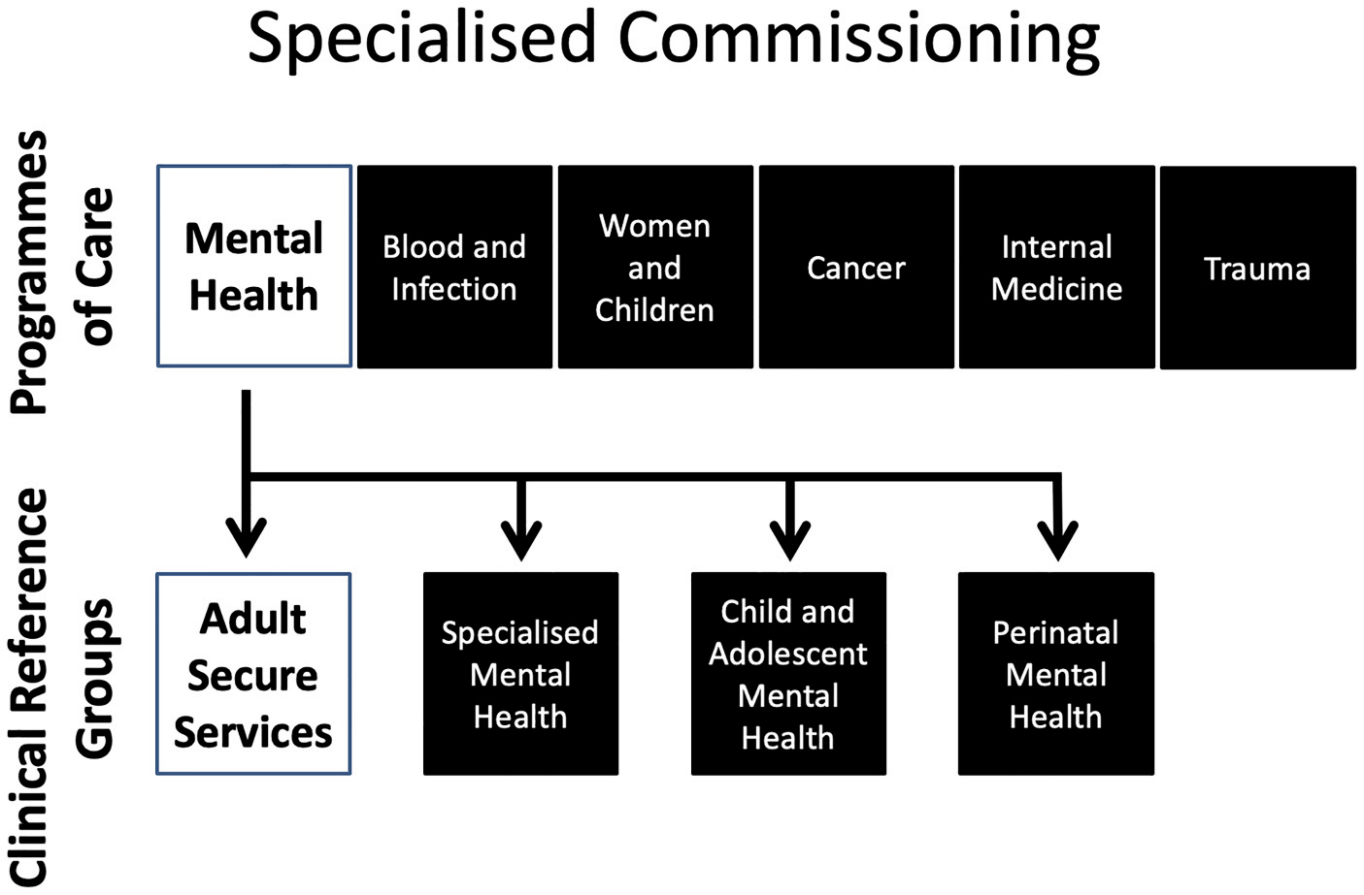
Diagrammatic representation of the structure of the commissioning of specialised services at NHS England and NHS Improvement.

Adult secure services in England are directly planned and funded by NHSEI through specialised commissioning, although some of these responsibilities are delegated to NHS-led Provider Collaboratives for adult medium and low secure services, which currently exclude secure D/deaf, secure acquired brain injury and women's enhanced medium secure services. Specialised commissioning at NSHEI plans, agrees and monitors a range of services to support people with rare and complex conditions. These services are planned on a national basis to ensure that services match the specific population needs across the country, in contrast to more routine services, which are locally commissioned. Services that are centrally commissioned are divided in to six national so-called programmes of care, one of which is mental health, learning disability and autism. Each of these is advised by several CRGs, which provide clinical, professional and expert by experience advice and leadership (see Figure 1).

The CRG for Adult Secure Services covers high, medium and low secure, and some community forensic mental health services, for both men and women. The purpose of the CRG is to provide clinical advice and leadership to specialised services; including leading on policy development, creating quality standards for evaluating services; and reducing regional variation. It creates several tools to support commissioning, such as specifications setting out standards for secure services and guidance on particular topics, such as transitions from adolescent to adult secure services, and managing a health weight in adult secure services.^18^

The CRG identified a lack of high-quality evidence available to guide much of its activities and set out to establish priority areas in which research is needed. The aim was to systematically identify relevant research questions, prioritise these and then work with other organisations to develop the research necessary to answer them. This focused specifically on questions related to the effective functioning of forensic mental health services as a whole, rather than on the efficacy of individual therapeutic interventions. The first step was to establish a research subgroup to determine the required approach and generate possible research questions. This subgroup contained members of the CRG, clinicians from a range of disciplines, commissioners, academics, and experts by experience, including those with experience as patients in forensic mental health services, and their families and carers. Members of the subgroup and the wider CRG membership were invited to submit ideas for potential research questions. To ensure coverage of all relevant service areas, representatives from high secure, women's, and community forensic services were also asked to contribute ideas. Over forty different research questions were identified through this process. To prioritise these further, they were grouped thematically into seven related areas, namely outcomes, involvement and experience, pathways of care, risk assessment, workforce and infrastructure, tackling inequalities, and treatment options. These were presented to the CRG membership, which was asked to vote on the relative importance of each.

The leads for the research subgroup were then able to use this to inform discussions with colleagues from the National Institute for Health Research (NIHR) to determine how these areas aligned with wider priorities for mental health research.^19^ It also offered the opportunity to establish which research questions were already being investigated by checking these against the NIHR's national portfolio of funded projects. This resulted in two areas being selected for further development:

1. Evaluation of the process of access assessment for medium and low secure services, which is used to determine whether it is appropriate to admit a patient to a secure hospital and, if so, the level of security warranted. This includes an examination of the use of structured professional judgement tools to guide access assessments. This research is needed to provide a solid evidence base to inform effective decision-making so that patients are correctly placed in the most appropriate service for them, while improving the robustness of decision making and reducing geographical variability.
2. The need for a greater understanding about the process of collaborative risk assessment across all levels of security, whereby patients are involved as partners with the clinical team in understanding and managing their risk. While this approach has been advocated for some time and areas of good practice exist, there remains little evidence that it has been widely implemented.^20^

The first area was subsequently developed into a proposal to the Research Needs Panel at NHS England. The Research Needs Panel was established in 2014 to evaluate gaps in the evidence identified by teams in NHS England and promote the use of evidence-based decision making. This submission was led by the authors of this paper who include commissioners and clinicians, representing the CRG, the research sub-group and Provider Collaboratives. The proposal subsequently progressed through an iterative process to the UK Department of Health and Social Care, which agreed to commission the research through an open call.^21^ The plan is to follow suit with the second idea, once work on the first is underway.

## Reflections on the process

Overall, this was a positive experience in many ways. The excellent engagement from all stakeholders demonstrates that clinicians, commissioners, experts by experience and academics are interested in driving the research agenda forward. Co-production of the proposal from the outset ensured that proposals address questions of true clinical importance. The newly established Adult Secure Provider Collaborative network, which spans medium and low secure inpatient services, offers the potential to access local clinical, expert by experience and commissioning expertise, and to disseminate results and implement learning from the research. The process was also able to benefit from access to high secure membership through the CRG, and the Clinical Secure Practice Forum for high secure services (a subgroup to the CRG). This demonstrated the effectiveness of the CRG as a nexus for coordinating the identification and prioritisation process, involving all relevant stakeholders by drawing on its well-established networks. Its position within NHS England and NHS Improvement also enabled it to access the internal Research Needs Panel, which is specifically designed to respond to this type of evidence deficit.

The process required careful liaison with different teams both within NHSEI and external partners such as the NIHR and Care Quality Commission, to ensure that all relevant parties were involved and to prevent unnecessary duplication of effort. The timescales have been considerable, taking around two years from the initial decision to form the research subgroup to the commissioning of the first piece of research. This has meant that there has been limited opportunity to respond to potentially changing priorities, particularly in light of the current global pandemic.^22^

The Adult Secure Services CRG brings several important stakeholders together, including clinicians from different professional groups, experts by experience (both patients and their families), public health experts, and professional associations. It is therefore well-placed to ensure that attention is paid to translational research in forensic mental health settings, so that the output of research influences clinical policy. Research into new treatments and therapies for mental illnesses is crucial, of course; but so is research into the efficacy and effectiveness of processes and instruments used routinely in clinical practice, and the CRG is uniquely well-placed to facilitate this.

The approach taken by the CRG, whilst it undoubtedly brings benefits, also has some potential drawbacks. Prioritisation of certain systems-based topics for such research funding may cause concern that other areas of equal clinical importance, such as novel treatments or forms of rehabilitation, are being neglected. However, this is not a ‘zero sum game’ and there is a need to advocate for greater resource to be allocated to research in all areas of mental health delivery and practice.^23^ It is also important that research is led by clinicians and experts by experience working together in partnership. There is a risk that if research goes out to tender that it will be performed by non-clinician collaboratives, which do not understand the area.^24^ It is therefore vital that any future model for commissioning research includes clinicians and experts by experience throughout the life cycle of the research, from conceptualisation to dissemination.

## Vision for the future

Historically mental health services have often been neglected when establishing policy research priorities. Our experience has demonstrated the need to establish a more systematic mechanism for identifying research priorities that matter to frontline mental health services, working with stakeholders to develop these ideas and advancing them to be funded as discrete pieces of research. Commissioners are ideally placed to act as the fulcrum for this work, through their network of providers, who can identify research questions from their direct clinical experience. The new commissioning framework utilising the NHS-led Provider Collaboratives for adult medium and low secure services supports this process and allows frontline providers to take a greater lead role in the future. Commissioners potentially also have access to sources of funding through centralised channels, such as the Research Needs Panel, although this would benefit from further streamlining. The process of identifying research needs should be dynamic, so that priorities are reviewed regularly to ensure that they are still important and have not been addressed by other research initiatives in the meantime. Experts by experience must be routinely involved from the outset so that research is better tailored to reflect the needs of service users, their families and carers.^12^ Alongside these mechanisms for identifying priorities, urgent capacity building is needed to enhance the clinical academic capabilities in forensic mental health necessary to deliver impactful research.

We believe that improving the process for commissioning research in this way could offer a mechanism by which health services research in forensic mental health services could be promoted and strengthened.
